# Conduction Mechanisms on High Retention Annealed MgO-based Resistive Switching Memory Devices

**DOI:** 10.1038/s41598-018-33198-0

**Published:** 2018-10-03

**Authors:** D. J. J. Loy, P. A. Dananjaya, X. L. Hong, D. P. Shum, W. S. Lew

**Affiliations:** 1 School of Physical and Mathematical Sciences, Nanyang Technological University, 21 Nanyang Link, Singapore, 637371 Singapore; 2 Globalfoundries Singapore Pte Ltd, 60 Woodlands Industrial Park D Street 2, Singapore, 738406 Singapore

## Abstract

We report on the conduction mechanisms of novel Ru/MgO/Cu and Ru/MgO/Ta resistive switching memory (RSM) devices. Current-voltage (I–V) measurements revealed Schottky emission (SE) as the dominant conduction mechanism in the high resistance state (HRS), which was validated by varying temperatures and transmission electron microscopy (TEM) results. Retention of more than 10 years at 85 °C was obtained for both Ru/MgO/Ta and Ru/MgO/Cu RSM devices. In addition, annealing processes greatly improved the consistency of HRS and LRS switching paths from cycle to cycle, exhibiting an average ON/OFF ratio of 10^2^. Further TEM studies also highlighted the difference in crystallinity between different materials in Ru/MgO/Cu RSM devices, confirming Cu filament identification which was found to be 10 nm in width.

## Introduction

Over the past decade, non-volatile memories research has been on the surge in institutions and industries to improve the endurance and reliability of flash memories. These limitations have become more significant as memory devices scale down in sizes to keep up with the rising demands of the digital world^1^. In recent years, alternative NVMs^2^ such as resistive random access memories (RRAMs) have emerged as one of the potential candidates to replace flash memories, owing to its simple metal-insulator-metal (MIM) structure, complementary metal oxide semiconductor (CMOS) compatibility, fast access speeds and low power consumptions^3–5^.

Since then, numerous RRAM insulator materials such as binary metal oxides^6,7^, chalcogenides^8,9^ and perovskite oxides^10,11^ have been studied. However, among these materials, binary metal oxides were the most extensively studied due to their resistance to thermal stress and compatibility with CMOS^5–7^. These characteristics make binary metal oxides suitable for memory devices and one such binary metal oxide is magnesium oxide (MgO). MgO is a potential candidate for the insulating layer of RRAM due to intrinsic properties such as large bandgap, high dielectric constant and high thermal conductivity^12,13^. According to the MgO-based RRAM works published in literature, inert electrodes Ru^14^ and Pt^12,15,16^ and active electrodes Ti^14^, ITO^17^, Pt^12,15^, Ta^16^ have been explored.

RRAM physics is an area where knowledge and understanding has been rather limited, and this led to the motivation of investigating RRAM physics in this work. The physics of RRAMs would involve investigations of conduction mechanisms and switching mechanisms. There are many types of conduction mechanisms in dielectric films, i.e. electrode-limited and bulk-limited conduction mechanisms. While Schottky emission (SE)^18–20^, Fowler-Nordheim (F-N) tunnelling^21,22^, direct tunnelling^23,24^ and thermionic-field emission^25,26^ are electrode-limited conduction mechanisms, Poole-Frenkel (P-F) emission^27,28^, hopping conduction^29,30^, Ohmic conduction^12,31^, space-charge-limited-conduction (SCLC)^32,33^, ionic conduction^34,35^, and trap-assisted tunnelling (TAT)^4,36^ are bulk-limited conduction mechanisms^24^. In this work, conduction mechanisms were investigated from current-voltage (I–V) graphs of RRAM devices, validated by both temperature variation and transmission electron microcopy (TEM) studies.

**Figure 1:**
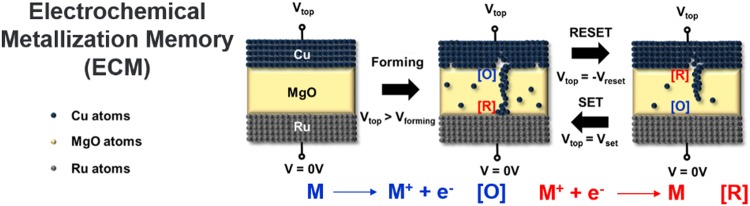
Electrochemical metallization (ECM) schematic process in Ru/MgO/Cu RSM devices. During the SET process, positive voltage (larger than forming voltage) is applied to the top electrode to oxidize Cu into Cu^+^ ions at the Cu/MgO interface [O]. Cu^+^ ions were reduced at the Ru/MgO interface to form back Cu atoms [R]. This process continues until the entire filament is formed. The RESET process would require a negative voltage applied to the top electrode to oxidize Cu atoms into Cu^+^ ions at the Ru/MgO interface [O]. Cu^+^ ions were reduced at the Cu/MgO interface to form back Cu atoms [R], thus rupturing the filament.

The most common switching mechanisms in RRAMs are known to be valence change memory (VCM) and ECM memory. Switching mechanisms of RRAMs are determined by the type of filament formed between the active and inert electrodes of the RRAM. While filaments in VCM are formed by anionic oxygen vacancies, filaments in ECM are formed by cationic species generated from the active electrode. For simplicity, the schematic of filament evolution in Ru/MgO/Cu RSM ECM devices was exhibited in Fig. 1. The rupturing and forming of filament give rise to binary memory states of RRAM devices determined by its high resistance state (HRS) and low resistance state (LRS) respectively^37^. A SET state occurs when HRS becomes LRS while a RESET state occurs when LRS becomes HRS^38^. In the SET process, a compliance current limit is imposed to protect the resistive switching memory (RSM) devices from high current damages^5^. As exhibited in Fig. 1, during the SET process, a positive voltage (larger than forming voltage) is applied to the top electrode to oxidize Cu into Cu^+^ ions at the Cu/MgO interface. Cu^+^ ions were reduced at the Ru/MgO interface to form back Cu atoms. This process continues until the entire filament is formed. Similarly, the RESET process would require a negative voltage applied to the top electrode to oxidize Cu atoms into Cu^+^ ions at the Ru/MgO interface. Cu^+^ ions were reduced at the Cu/MgO interface to form back Cu atoms, thereby rupturing the filament^34^.

In this work, we initiated an investigation of a less commonly studied oxide material, i.e. MgO but has shown great potential in terms of physics and performances^12–17^. We also included Ru/MgO/Cu RSM devices in the investigation. As Ru/MgO/Cu ECM RSM devices have not been reported before in literature, it would be meaningful to compare its performances with other reported VCM MgO-based RSM devices. The investigation of ECM Ru/MgO/Cu RSM devices would open the doors for filament evolution studies. Conduction mechanisms in the Ru/MgO RSM device interface were also investigated for Cu and Ta active electrodes. The conduction mechanisms investigated in this work were predominantly Schottky emission (SE) and Fowler-Nordheim (FN)-tunnelling as explained in the Results and Discussions section.

## Results and Discussion

**Figure 2:**
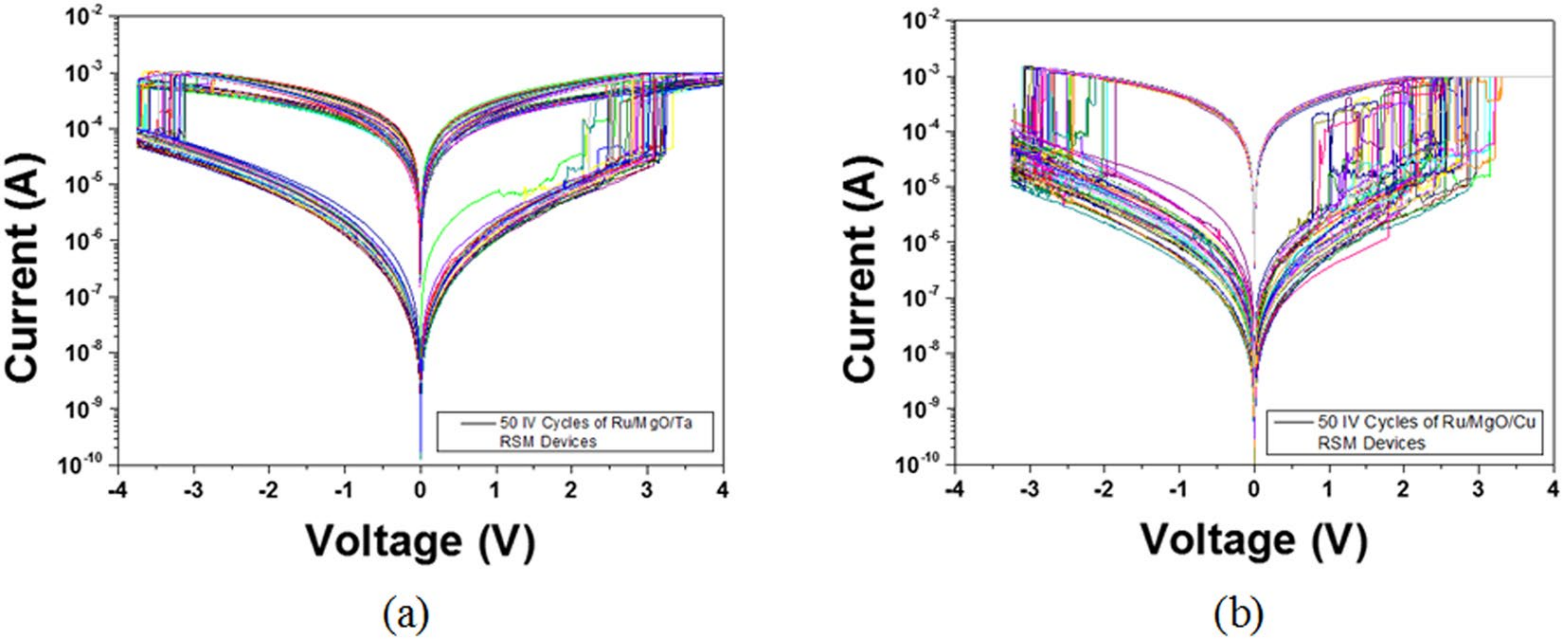
50 semi-log IV cycles of (**a**) Ru/MgO/Ta and (**b**) Ru/MgO/Cu RSM devices. It can be observed that the switching voltage distribution of Ru/MgO/Ta RSM devices was found to be more consistent than Ru/MgO/Cu RSM devices. The average ON/OFF ratio of the RSM devices was found to be 10^2^.

**Figure 3:**
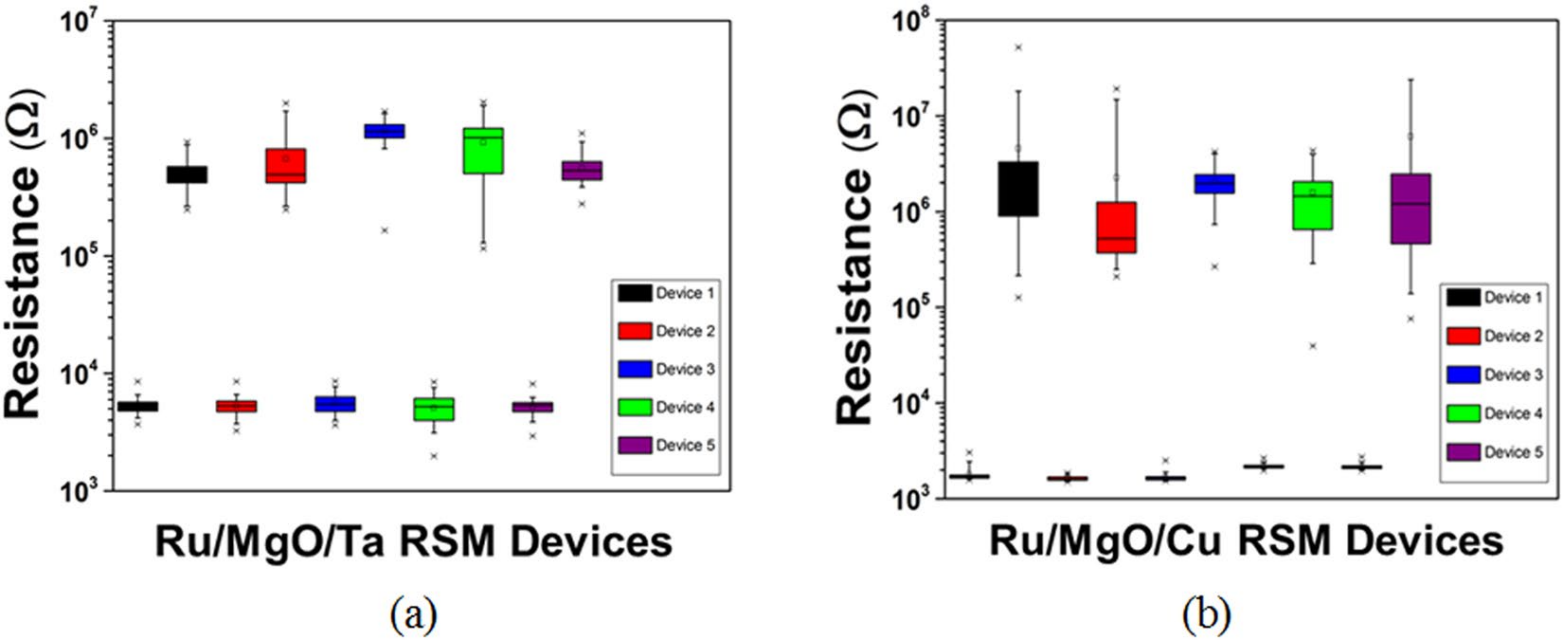
Box plots of 5 (50 cycles each) (**a**) Ru/MgO/Ta and (**b**) Ru/MgO/Cu RSM devices. Ru/MgO/Ta RSM devices exhibited a much better distribution and less variation in terms of HRS as compared to Ru/MgO/Cu RSM devices. This could be attributed to the lower diffusivity effect of Ta atoms in Ru/MgO/Ta RSM devices, resulting in a better control of filament formation/rupture.

In the investigation of conduction mechanisms in MgO-based RSM devices, single 1R cells with dimensions 30 µm × 30 µm were characterized, as shown in Figs 2 and 3. 50 cycles of semi log I–V curves of Ru/MgO/Ta and Ru/MgO/Cu RSM devices were exhibited in Fig. 2(a,b) respectively. It was observed that the switching voltage distribution of Ru/MgO/Ta RSM devices was found to be more consistent than Ru/MgO/Cu RSM devices. The average ON/OFF ratios of Ru/MgO/Ta and Ru/MgO/Cu RSM devices were found to be 10^2^. Box plots for 5 devices of 50 cycles each for Ru/MgO/Ta and Ru/MgO/Cu RSM devices were shown in Fig. 3(a,b) respectively. As seen from Figs 2 and 3, Ru/MgO/Ta resistive switching memory (RSM) devices have a much tighter high resistance state (HRS) distribution as compared to Ru/MgO/Cu RSM devices. This could be explained by the higher diffusivity effect of Cu as compared to Ta atoms into the MgO dielectric layer, which results in a larger variation of filament formation/rupture for Ru/MgO/Cu RSM devices as compared to Ru/MgO/Ta RSM devices. The variation of filament formation/rupture in turn results in a greater HRS variation for Ru/MgO/Cu RSM devices as compared to Ru/MgO/Ta RSM devices. The same reasoning can be used to explain the larger switching voltage variation in Ru/MgO/Cu RSM devices as compared to Ru/MgO/Ta RSM devices.

**Figure 4:**
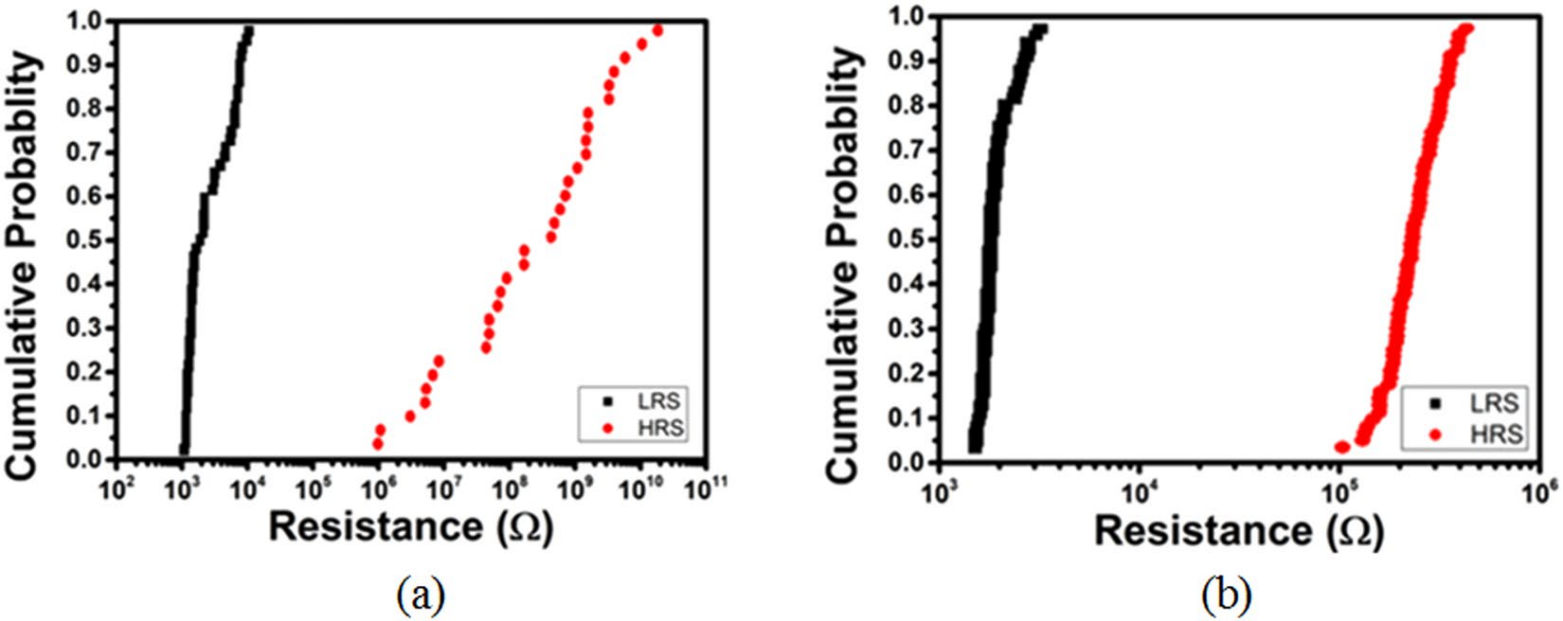
Statistical cycle-to-cycle resistance variations between **(a)** non-annealed and **(b)** annealed Ru/MgO/Ta RSM devices. It is evident that despite a lower ON/OFF ratio, annealed RSM devices exhibit less cycle-to-cycle resistance variation as compared to annealed ones.

The statistical cycle-to-cycle resistance variations of non-annealed and annealed RSM devices were compared in Fig. 4(a,b) respectively. It was shown that despite having a lower ON/OFF ratio of annealed RSM devices, they can exhibit better cycle-to-cycle resistance variations as compared to non-annealed RSM devices. The low variation in cycle-to-cycle resistance is imperative to conduction mechanism studies because filament cross-sectional area should be kept as consistent as possible. Keeping the filament cross-sectional area constant improves the quality of conduction mechanism. This minimizes the variables affecting the conduction mechanism process as the temperature would be varied.

Prior to performing the conduction mechanism fittings, the conduction mechanism for the MgO-based RSM was suggested to be an electrode-limited type rather than bulk-limited type. This can be attributed to Ru being the inert electrode for the MgO-based RSM devices. It was reported that the conduction band offset^7^ ΔE_C_ between MgO and Si is 1.5 eV, while the electron affinity^22^ of Si and work function^39^ of Ru are 4.05 eV and 4.71 eV respectively. This means that the ΔE_C_ between MgO and Ru has to be 2.16 eV, which is about 1 eV lower than the energy barrier between Pt and MgO as reported^12^. While the Pt/MgO barrier is a bulk-limited conduction mechanism due to the high energy barrier, the Ru/MgO interface should be dominated by electrode-limited conduction mechanism due to its lower energy barrier. In this work, it would be shown that electrode-limited conduction mechanism is dominant in Ru/MgO/Cu and Ru/MgO/Ta RSM devices.

The first conduction mechanism investigated was Schottky emission (SE), which takes place when thermally activated electrons gained enough energy to jump into the conduction band of the dielectric layer. SE is the most observed conduction mechanism and is an electrode-limited conduction mechanism. Equation (1) exhibits the current density expression for SE^24,40^ where q is the electron charge, m^*^ is the electron effective mass in the oxide, k is Boltzmann’s constant, T is the absolute temperature, h is Planck’s constant, Φ_*B*_ is the junction barrier height, E is the electric field across the oxide, and ε is the permittivity of the oxide.

1 $${{\rm{J}}}_{{\rm{SE}}}=\frac{{4{\rm{\pi }}\mathrm{qm}}^{\ast }{(\mathrm{kT})}^{{\rm{2}}}}{{{\rm{h}}}^{{\rm{3}}}}\exp \,[\frac{-\,{\rm{q}}({{\rm{\Phi }}}_{{\rm{B}}}-\sqrt{\mathrm{qE}/4{\rm{\pi }}{\rm{\varepsilon }}})}{{\rm{kT}}}]$$

FN-tunnelling was investigated as another form of electrode-limited conduction mechanism. It occurs in high electric fields where the thickness of the dielectric is more than 3 nm, which fits the criteria of the investigated MgO layer thickness. Equation (2) exhibits the current density expression for FN-tunnelling^24,40^ and the parameters used in equation (2) are similar to those used in equation (1).

2 $${{\rm{J}}}_{{\rm{FN}}}=\frac{{{\rm{q}}}^{{\rm{2}}}}{{\rm{8}}\pi {{\rm{h}}{\rm{\Phi }}}_{{\rm{B}}}}{{\rm{E}}}^{{\rm{2}}}\exp [\frac{-8{\rm{\pi }}\sqrt{{{\rm{2qm}}}^{\ast }}}{{\rm{3hE}}}{{{\rm{\Phi }}}_{{\rm{B}}}}^{3/2}]$$

**Figure 5:**
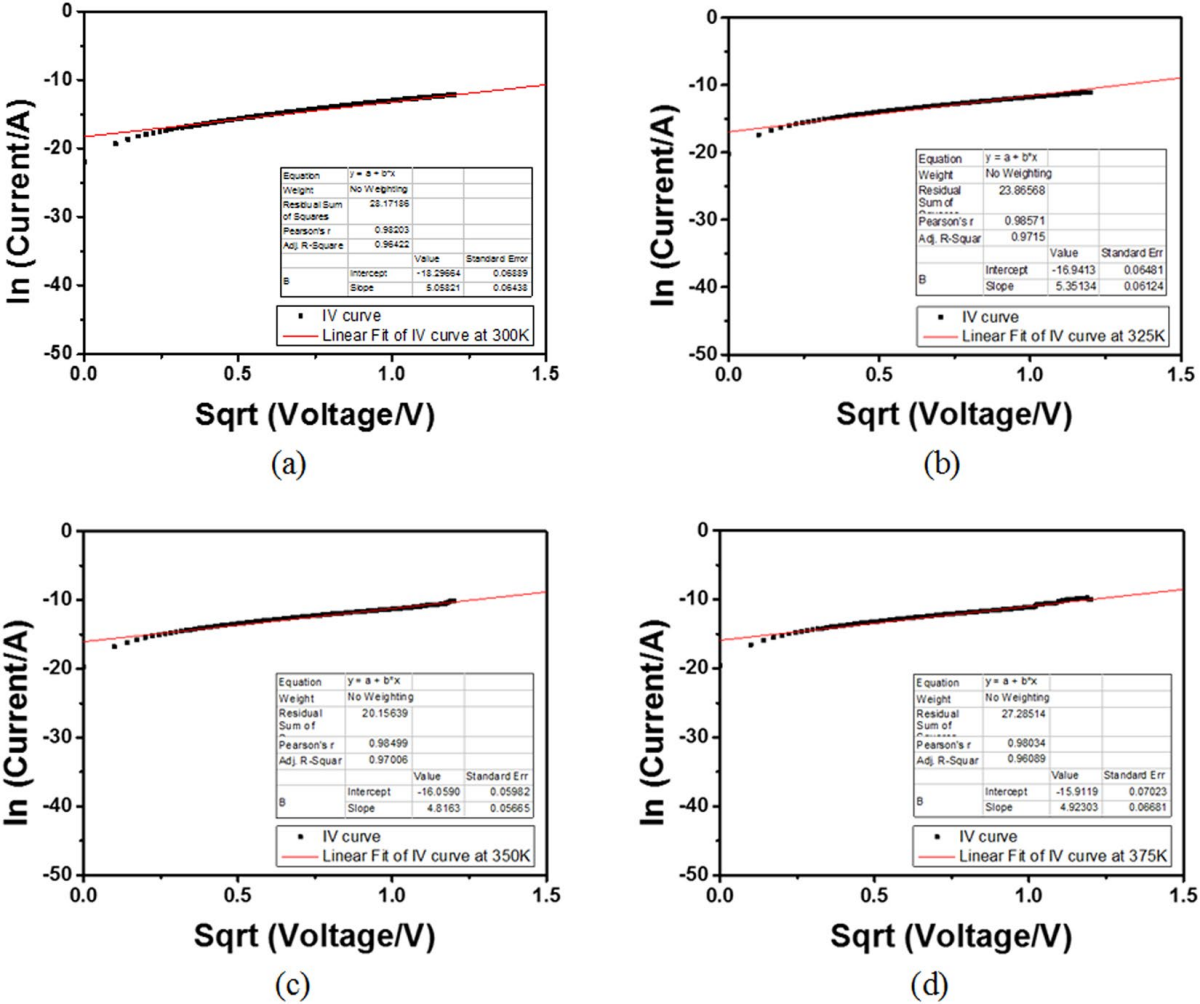
ln(current) vs. sqrt(voltage) and linear fitting for Ru/MgO/Ta RSM devices at (**a**) 300 K, (**b**) 325 K, (**c**) 350 K, (**d**) 375 K The linear fitting shows that Ru/MgO/Ta RSM devices exhibit SE.

In the investigation of SE, the temperature was varied from 300 K to 375 K in intervals of 25 K while I–V curves were characterized. It is worthy to note that the cross-sectional area of the filament was kept constant by applying a voltage less than the switching voltage of the RSM devices. Equation (3) shows the SE fitting curve of the form $\mathrm{ln}({{\rm{I}}}_{{\rm{SE}}}){\rm{vs}}{\rm{.}}\sqrt{{\rm{V}}}$ which in turn generated the slope, M for each of the stated temperatures (300 K, 325 K, 350 K and 375 K) as shown in Fig. 5(a–d) respectively. The linear fit shows that Ru/MgO/Ta RSM devices exhibit SE conduction mechanism. TEM was used to further verify the SE conduction mechanism by comparing the actual and calculated values of d, the distance between the tip of the filament and the MgO/Ru interface. d can be calculated from M as shown in equation (5). The values of M and d are shown in Table 1.

3 $$\mathrm{ln}({{\rm{I}}}_{{\rm{SE}}})=\,\mathrm{ln}(\frac{{4{\rm{\pi }}\mathrm{qm}}^{\ast }{(\mathrm{kT})}^{{\rm{2}}}{\rm{A}}}{{{\rm{h}}}^{{\rm{3}}}})+\frac{-\,{{\rm{q}}{\rm{\Phi }}}_{{\rm{B}}}}{{\rm{kT}}}+\frac{{\rm{q}}\sqrt{{\rm{q}}/{\rm{4}}\pi \varepsilon {\rm{d}}}}{{\rm{kT}}}\sqrt{{\rm{V}}}$$

4 $${\rm{M}}=\frac{\sqrt{{{\rm{q}}}^{{\rm{3}}}{/{\rm{\pi }}{\rm{\varepsilon }}}_{{\rm{r}}}{{\rm{\varepsilon }}}_{{\rm{0}}}{\rm{d}}}}{{\rm{2kT}}}$$

5 $${{\rm{d}}}_{{\rm{SE}}}=\frac{{{\rm{q}}}^{{\rm{3}}}}{{(\mathrm{2kTM})}^{{\rm{2}}}{\rm{\pi }}{\rm{\varepsilon }}}$$

**Table 1:** Relationship between temperature, T, gradient of linear fit, M and the distance between the tip of the filament and the MgO/Ru interface, d for Ru/MgO/Ta devices.

T/K	M	d/m
300	5.06	8.84 × 10^−9^
325	5.35	6.73 × 10^−9^
350	4.81	7.18 × 10^−9^
375	4.92	5.98 × 10^−9^

The general trend shows that with an increase in temperature, d decreases.

**Figure 6:**
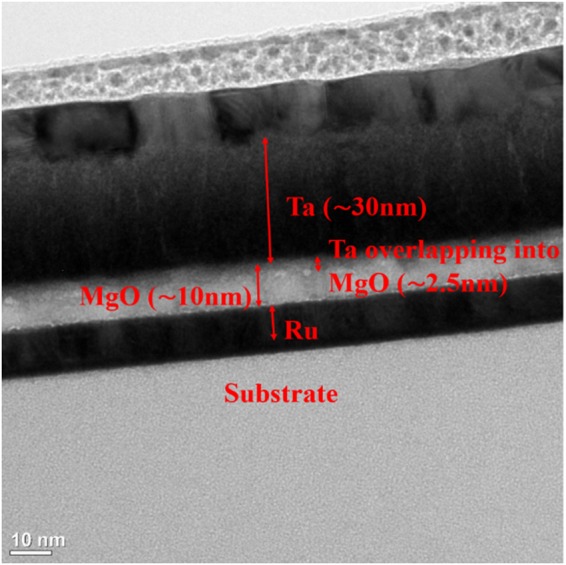
TEM image of Ru/MgO/Ta RSM device. This image shows the different layers of the RSM device discussed in this work. TEM was employed to investigate the thickness of MgO and it was observed that a thin layer of Ta of ~2.5 nm was diffused into MgO. This validates that the conduction mechanism for the Ru/MgO/Ta RSM devices is SE, as the actual distance between the tip of the filament and the MgO/Ru interface, d for Ru/MgO/Ta devices corresponds to the order of d values shown in Table 1.

In Table 1, it can be generally concluded that with an increase in temperature, the distance d decreases. This coincides with the fact when d increases (decreases), the HRS resistance increases (decreases) proportionally, as more (less) filament is being ruptured. A TEM image of the Ru/MgO/Ta RSM device as exhibited in Fig. 6 where a thin layer of ~2.5 nm Ta was diffused into MgO. The diffusivity of Ta into MgO was attributed to the annealing process which agitates the Ta/MgO interfacial atoms, causing the Ta atoms to cross over the grain boundaries of MgO atoms. It can be concluded that the diffusivity of Ta into MgO had resulted in a lower thickness of MgO (actual d = 7.5 nm vs. calculated d = 8.84 nm at 300 K) and further validated the SE conduction mechanism of the Ru/MgO/Ta RSM device.

**Figure 7:**
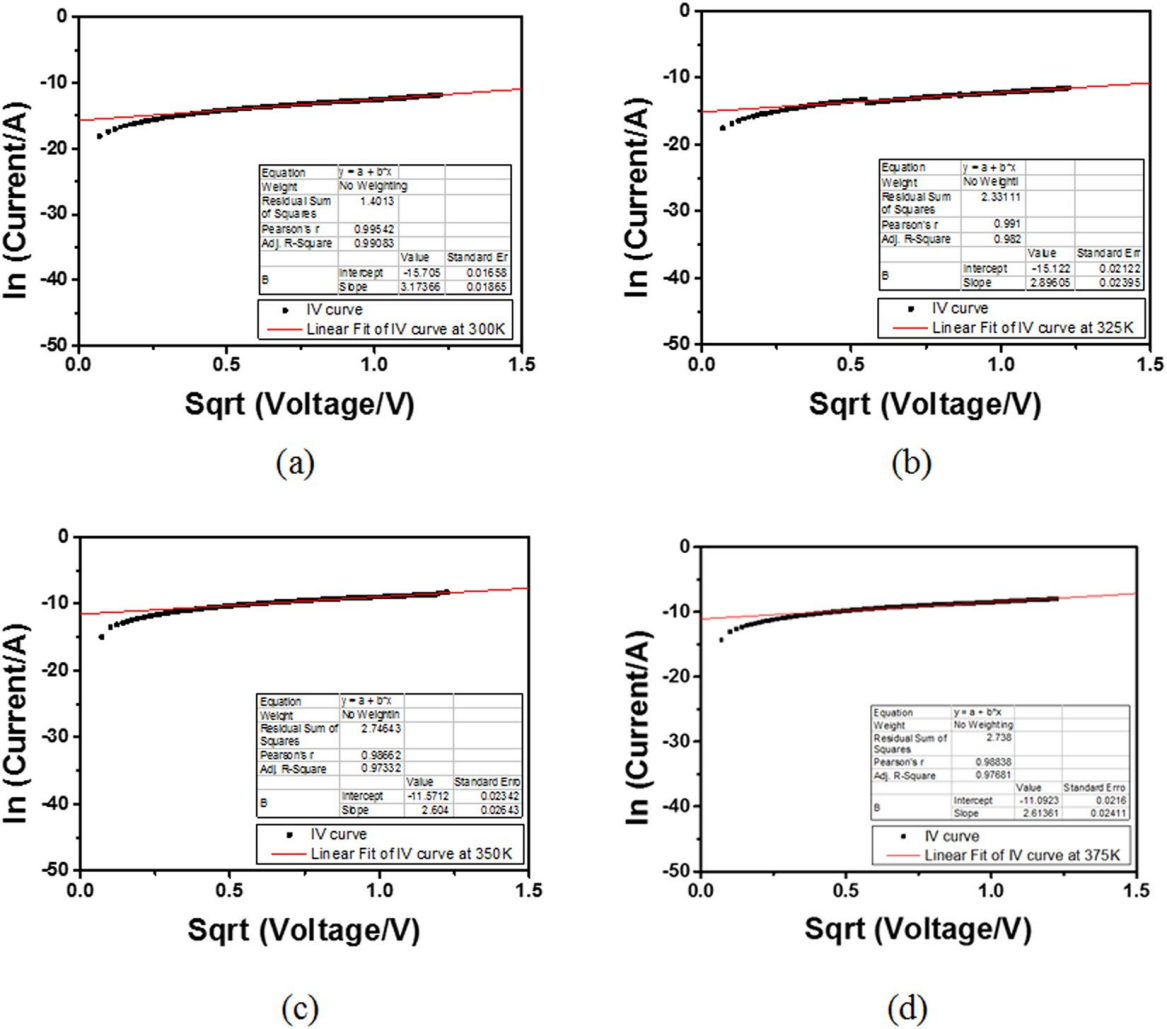
ln(current) vs. sqrt(voltage) and linear fitting for Ru/MgO/Cu RSM devices at (**a**) 300 K, (**b**) 325 K, (**c**) 350 K, (**d**) 375 K. The linear fitting shows that Ru/MgO/Cu RSM devices also exhibit SE.

The same characterization and analysis was performed for Ru/MgO/Cu RSM devices. The same conclusions can be made about Ru/MgO/Cu RSM devices as shown in Fig. 7(a–d) where it was shown that SE is also the main conduction mechanism. Similar analysis and discussions exhibited that FN-tunnelling conduction mechanism was rejected as the main conduction mechanism for the RSM devices due to the poorly fitted curves shown the Supplementary information S1(a–d).

**Figure 8:**
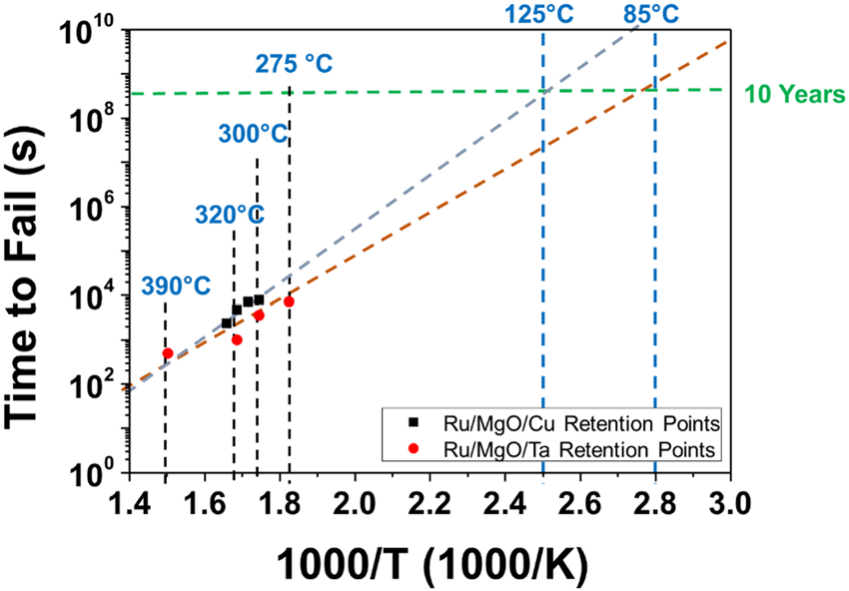
Retention data of Ru/MgO/Cu and Ru/MgO/Ta RSM devices were obtained using *in-situ* temperature stress. The Arrhenius plot (time to fail vs. 1000/T) was extrapolated, where T is the temperature in Kelvins. The temperatures were varied and their respective times to fail were selected for the retention plot. An extrapolation shows that the Ru/MgO/Cu and Ru/MgO/Ta RSM devices were able to retain its memory for more than 10 years at 85 °C.

Aside from investigating the physics, it is also important to investigate the data retention of the RSM devices. Ru/MgO/Cu and Ru/MgO/Ta RSM devices were heated using various high temperatures to accelerate the rate of failure. At the same time, the devices were characterized to obtain their resistance values at fixed intervals of time. The retention data were plotted in Fig. 8 and was fitted using Arrhenius equation^41^ where the time to fail (lifetime) for each RSM device was plotted against the reciprocal of the heated temperature. The lifetime was obtained from Arrhenius equation shown in equation (8), where B is a pre-exponential factor the accounts for the frequency of atomic collisions and orientations, E_a_ the activation energy of the reactions in the RSM devices, k the Boltzmann constant while T is the absolute temperature supplied to the system.

6 $${\rm{Lifetime}}={\rm{B}}\cdot \exp (\frac{{{\rm{E}}}_{{\rm{a}}}}{{\rm{kT}}})$$

An extrapolation of the data points shows that both Ru/MgO/Cu and Ru/MgO/Ta RSM devices were able to retain its memory for more than 10 years at 85 °C. Ru/MgO/Cu RSM devices performed better as compared Ru/MgO/Ta RSM devices as they were able to exhibit a longer retention time or lifetime. It was also found from equation (8) that the E_a_ for the redox reactions during the retention process of Ru/MgO/Ta RSM devices was 0.7 eV. This value is very close to the activation energy for the retention process of flash memories of 0.6 eV^41^, which shows that the retention of Ru/MgO/Ta RSM devices fabricated in this work is comparable to the retention capabilities of flash memories. Similarly, the E_a_ of Ru/MgO/Cu RSM devices was calculated to be 1.17 eV. However, it is believed that the pre-exponential factor, B varies with different types of insulators and electrode materials, thus producing different E_a_ in the process. Hence, an RSM with a higher E_a_ does not directly represent a better retention, even though it generally suggests so.

TEM studies were also performed to investigate the filament formation in Ru/MgO/Cu RSM devices. These studies were performed in Ru/MgO/Cu rather than Ru/MgO/Ta RSM devices as copper filaments in ECM Ru/MgO/Cu RSM devices are more easily observed as compared to oxygen vacancies in VCM Ru/MgO/Ta RSM devices. In the filament analysis of Ru/MgO/Cu RSM devices, 0.5 µm × 0.5 µm devices were specially designed via electron beam lithography (EBL). A smaller RSM device size of 0.5 µm × 0.5 µm was used for the purpose of concentrating and confining filaments to a small region.

**Figure 9:**
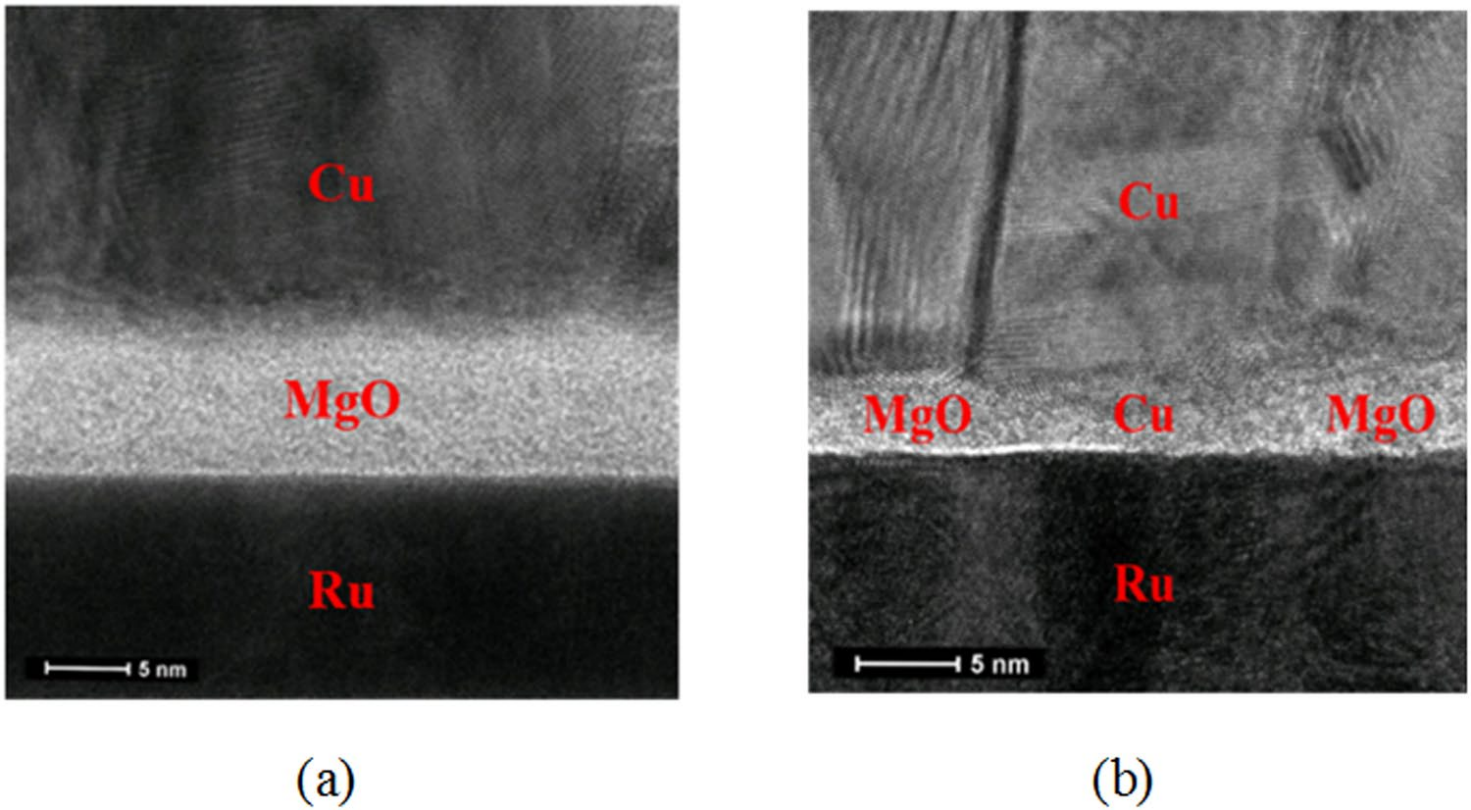
(**a**) TEM image of pristine Ru/MgO/Cu, **(b)** TEM image of SET state Ru/MgO/Cu. The TEM image shown in (**b**) was obtained after applying a voltage of 2 V. It is believed that the central region of the MgO layer shown in (**b**) is a Cu filament, due to similar crystallinity exhibited by Cu atoms shown in the TEM image. In addition, it was also observed that the average width of the filament is about 10 nm.

The TEM images of pristine and SET state Ru/MgO/Cu RSM devices were shown in Fig. 9(a,b) respectively, where the TEM image exhibited in Fig. 9(b) was obtained after a voltage of 2 V was applied. Observing the insulator layer in Fig. 9(b), the region concentrated at the centre consists of crystalline atoms belonging to Cu atoms, while the regions away from the centre consists MgO atoms which are less crystalline (or amorphous). With this observation, it can be concluded that the crystalline atoms located at the centre belongs to Cu atoms, thus making up the Cu filament. It was also observed that the width of the filament is about 15 nm from the MgO/Cu interface, with a decreasing width to 5 nm at the Ru/MgO interface, which averaged to be about 10 nm.

In this work, we investigated the annealing effect on cycle-to-cycle resistance variability, conduction mechanisms, retention and the presence of filaments of Ru/MgO/Ta and Ru/MgO/Cu RSM devices. The investigation of annealing effect on RSM devices is imperative to improve the cycle-to-cycle resistance variability, and in turn produce consistent resistance paths for conduction mechanism studies. In the characterization of Ru/MgO/Ta and Ru/MgO/Cu RSM devices, the average ON/OFF ratios obtained from their I–V curves were 10^2^. Conduction mechanism fittings were performed with varying temperatures of (300 K, 325 K, 350 K and 375 K) as was found to exhibit schottky emission (SE). This was further validated by TEM where the actual distance between the tip of the filament and the MgO/Ru interface was found to be similar to the calculated values. Retention results of Ru/MgO/Cu and Ru/MgO/Ta RSM devices were investigated using Arrhenius equation by failing the devices at a certain temperature and duration. The Arrhenius plots were extrapolated was found that the retention of Ru/MgO/Cu and Ru/MgO/Ta RSM devices were both 85 °C for more than 10 years. TEM analysis was also performed on Ru/MgO/Cu RSM devices and the Cu filament was identified by the difference in crystallinity, of which the width of the filament was measured to be 10 nm. The conduction mechanism results and performance in this work has shown with great certainty that along with its binary oxide counterparts, MgO-based RSM devices have great prospects towards application-based embedded memories. The next step would be to further investigate the physics of filament evolution in Ru/MgO/Ag ECM RSM devices, as well as the conduction mechanisms of other variations of MgO-based RRAM structures.

## Methods

### Device Fabrication

Single Ru/MgO/Cu and Ru/MgO/Ta 1R cells with dimensions 30 µm × 30 µm were fabricated in this work. A DC magnetron sputtering process was first performed on a Ru target where a thin layer of bottom Ru electrode was deposited in Ar ambient at room temperature. The pressure of the Ar gas was 2mTorr at 20 sccm. The samples were next patterned via a shadow mask process. An RF magnetron sputtering process was performed on an MgO target where a thin MgO dielectric layer was deposited in Ar ambient at room temperature. The pressure of the Ar gas during the deposition was 1.5mTorr at 20 sccm. A DC magnetron sputtering process was performed on a Cu target in Ar ambient at room temperature. The pressure of the Ar gas during the deposition was 6.5mTorr at 20 sccm. Ru/MgO/Cu RSM devices were fabricated and their I–V graphs were characterised. Annealing was performed in vacuum conditions at 300 °C for a short period of time. Similar processes were used to fabricate Ru/MgO/Ta RSM devices.

### Characterisation

This test setup is performed using the Keithley semiconductor analyzer, where voltage is applied while current is measured. Compliance current is provided by clamping the current at maximum 1 mA, which can be configured from the characterization system. Conduction mechanism and retention measurements were performed using an *in-situ* heater to vary different temperatures while characterizing I–V curves. The retention measurements of the Ru/MgO/Cu and Ru/MgO/Ta RSM devices were characterised at high temperatures for a certain period until failure (this period is also known as lifetime). A device failure will be determined when there is an unintended switching and that the device could not be switched back to its original state. The relationship between the lifetime and temperature was exhibited using Arrhenius equation as shown in equation (6).

### TEM & Filament studies

Single 1R cells with dimensions 0.5 µm × 0.5 µm of Ru/MgO/Cu RSM devices were fabricated using the same fabrication processes as its 30 µm × 30 µm counterparts, except that electron beam lithography was used. The filament studies and analysis were performed using TEM where crystallinity between pristine and SET state atoms were compared.

## Electronic supplementary material

Supplementary Information

## Data Availability

The datasets generated during and/or analysed during the current study are available from the corresponding author on reasonable request.
