# Supplementary material for: Conduction Mechanisms on High Retention Annealed MgO-based Resistive Switching Memory Devices
Source: Sci Rep. 2018 Oct 3;8:14774. doi: 10.1038/s41598-018-33198-0 (PMC6170501; doi:10.1038/s41598-018-33198-0)
Supplement: Supplementary file 1 — Supplementary Information [file 41598_2018_33198_MOESM1_ESM.docx]

*Supplementary Information:*

**Conduction Mechanisms on High Retention Annealed MgO-based Resistive Switching Memory Devices**

**D. J. J. Loy**^1,2^**, P. A. Dananjaya**^1^**, X. L. Hong**^1^**, D. P. Shum**^2^**, W. S. Lew**^1^^[[1]](#footnote-1)^*

^1^School of Physical and Mathematical Sciences, Nanyang Technological University, 21 Nanyang Link, Singapore 637371

^2^Globalfoundries Singapore Pte Ltd, 60 Woodlands Industrial Park D Street 2, Singapore 738406


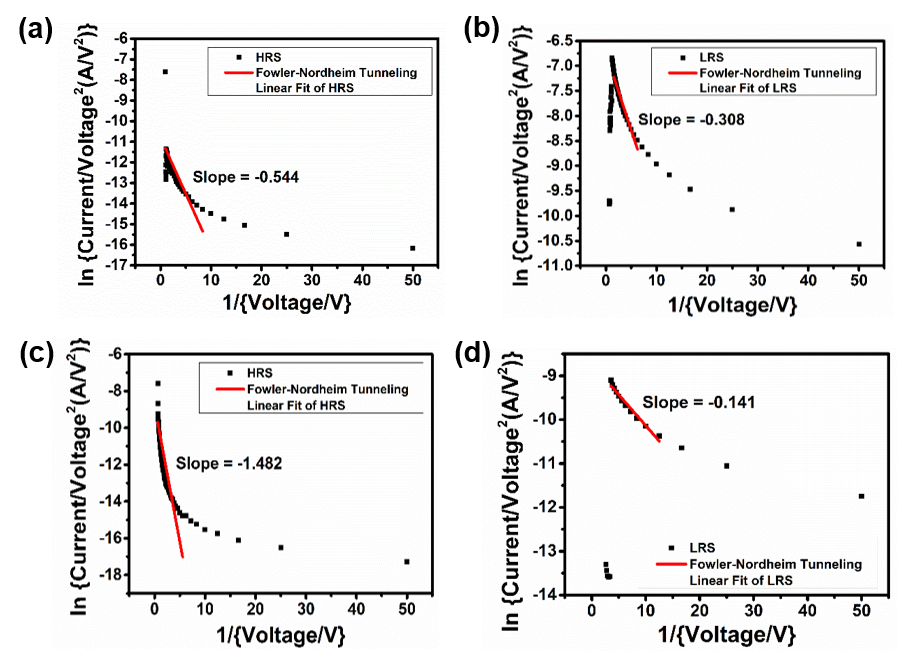


**Figure S1.**I/V^2^ vs. 1/V FN-tunnelling fit of **(a)** HRS Ru/MgO/Ta RSM device, **(b)** LRS Ru/MgO/Ta RSM device, **(c)** HRS Ru/MgO/Cu RSM device, **(d)** LRS Ru/MgO/Cu RSM device. As fitting is not linear, FN-tunnelling is regarded as not dominant conduction mechanism.

1. *Corresponding author: [wensiang@ntu.edu.sg](mailto:wensiang@ntu.edu.sg) [↑](#footnote-ref-1)
